# Prognostic significance of metabolomic biomarkers in patients with diabetes mellitus and coronary artery disease

**DOI:** 10.1186/s12933-022-01494-9

**Published:** 2022-05-07

**Authors:** Efstratios Karagiannidis, Dimitrios V. Moysidis, Andreas S. Papazoglou, Eleftherios Panteris, Olga Deda, Nikolaos Stalikas, Georgios Sofidis, Anastasios Kartas, Alexandra Bekiaridou, George Giannakoulas, Helen Gika, George Theodoridis, Georgios Sianos

**Affiliations:** 1 First Department of Cardiology, AHEPA University Hospital, Aristotle University of Thessaloniki, St. Kiriakidi 1, 54636 Thessaloniki, Greece; 2 Laboratory of Forensic Medicine and Toxicology, School of Medicine, Aristotle University of Thessaloniki, 54124 Thessaloniki, Greece; 3 Biomic_Auth, Bioanalysis and Omics Lab, Centre for Interdisciplinary Research of Aristotle, University of Thessaloniki, Innovation Area of Thessaloniki, 57001 Thermi, Greece; 4 Laboratory of Analytical Chemistry, Department of Chemistry, Aristotle University of Thessaloniki, Thessaloniki, Greece

**Keywords:** Diabetes mellitus, Metabolomic profiling, SYNTAX score, Coronary artery disease, Metabolomics biomarkers

## Abstract

**Background:**

Diabetes mellitus (DM) and coronary artery disease (CAD) constitute inter-related clinical entities. Biomarker profiling emerges as a promising tool for the early diagnosis and risk stratification of either DM or CAD. However, studies assessing the predictive capacity of novel metabolomics biomarkers in coexistent CAD and DM are scarce.

**Methods:**

This post-hoc analysis of the CorLipid trial (NCT04580173) included 316 patients with CAD and comorbid DM who underwent emergency or elective coronary angiography due to acute or chronic coronary syndrome. Cox regression analyses were performed to identify metabolomic predictors of the primary outcome, which was defined as the composite of major adverse cardiovascular or cerebrovascular events (MACCE: cardiovascular death, myocardial infarction, stroke, major bleeding), repeat unplanned revascularizations and cardiovascular hospitalizations. Linear regression analyses were also performed to detect significant predictors of CAD complexity, as assessed by the SYNTAX score.

**Results:**

After a median 2-year follow up period (IQR = 0.7 years), the primary outcome occurred in 69 (21.8%) of patients. Acylcarnitine ratio C4/C18:2, apolipoprotein (apo) B, history of heart failure (HF), age > 65 years and presence of acute coronary syndrome were independent predictors of the primary outcome in diabetic patients with CAD (aHR = 1.89 [1.09, 3.29]; 1.02 [1.01, 1.04]; 1.28 [1.01, 1.41]; 1.04 [1.01, 1.05]; and 1.12 [1.05–1.21], respectively). Higher levels of ceramide ratio C24:1/C24:0, acylcarnitine ratio C4/C18:2, age > 65 and peripheral artery disease were independent predictors of higher CAD complexity (adjusted β = 7.36 [5.74, 20.47]; 3.02 [0.09 to 6.06]; 3.02 [0.09, 6.06], respectively), while higher levels of apoA1 were independent predictors of lower complexity (adjusted β= − 0.65 [− 1.31, − 0.02]).

**Conclusions:**

In patients with comorbid DM and CAD, novel metabolomic biomarkers and metabolomics-based prediction models could be recruited to predict clinical outcomes and assess the complexity of CAD, thereby enabling the integration of personalized medicine into routine clinical practice. These associations should be interpreted taking into account the observational nature of this study, and thus, larger trials are needed to confirm its results and validate them in different and larger diabetic populations.

## Background

Diabetes mellitus (DM) and coronary artery disease (CAD) constitute two closely interacting clinical entities, whose concurrent prevalence is constantly rising [1–3]. DM is not only a major risk factor for the development of CAD, but is also associated with higher CAD complexity [4, 5], and worse clinical outcomes [6]. Apart from DM which has been always considered to have a metabolic substrate [7, 8] emerging evidence supports that the risk for CAD occurrence and progression is mediated by metabolism disturbances as well [9]. Concurrently, metabolomics is rapidly evolving as a novel technique of studying small molecule substrates, intermediates and products of cell metabolism [10]. This technique could be utilized to flag patients with higher risk for increased atherosclerotic burden, and subsequent future adverse clinical events [11].

Besides the already established biomarkers [12, 13], several other metabolomic and lipidemic indicators, such as ceramides, acylcarnitines, apolipoproteins and adiponectin, have been separately shown to increase the risk for CAD development and progression [14–17]. It has been proven that each of these biological intermediates of metabolism is involved in the atherosclerotic process, mostly by promoting cell apoptosis, coronary inflammation and plaque vulnerability. Furthermore, several metabolomic biomarkers have been shown to precisely identify patients being at high risk of developing DM, and to predict its major complications [18–22]. However, data concerning the metabolomic phenotyping of patients with CAD and comorbid DM are scarce. Moreover, there are almost no studies on this population aiming to use such data for the establishment of clinically useful indicators and models able to predict adverse clinical outcomes or the severity of CAD.

On the basis of the above, we conducted a retrospective analysis in a cohort of well-characterized patients with DM who underwent urgent or elective coronary angiography. Our aim was to investigate potential correlations between metabolic fingerprints and prospective major adverse cardiovascular or cerebrovascular events (MACCE) or baseline CAD complexity. This could lead to the development of predictive risk stratification algorithms for patients with CAD and comorbid DM.

## Methods

### Study design

This is an ancillary post-hoc analysis of the CorLipid trial (ClinicalTrials.gov identifier: NCT04580173) focusing on the metabolic profile of patients with DM and CAD. The protocol, study design and a subset of the main findings of the Corlipid trial have been previously published [23, 24]. In brief, CorLipid was a prospective, non-interventional cohort trial enrolling patients undergoing coronary angiography and aiming to assess the association of the patients’ metabolic profile with CAD severity [23].

The study protocol has been approved by the Scientific Committee of AHEPA University Hospital (reference number 12/13-06-2019) and every trial procedure was conducted according to the principles set by the declaration of Helsinki.

### Data sources

The data used in our study were extracted from the CorLipid database for the time range of July 2019 to May 2021. Medical history, baseline clinical characteristics, laboratory and echocardiographic data, as well as angiographic and metabolomic data were all included in the database. Follow-up data on clinical outcomes were also collected by trained independent investigators through telephonic or in-person interviews with the study participants.

### Study population

The population used for this analysis comprised of adult patients with either acute or chronic coronary syndrome (ACS or CCS) and comorbid DM type 2, who underwent elective or urgent coronary angiography in the cardiology department of an academic urban hospital (AHEPA University Hospital of Thessaloniki, Greece). Patients with prior coronary revascularization procedure or cardiopulmonary arrest upon admission were excluded. Patients with missing data concerning DM status and patients, who reported less than 8 h of fasting before coronary angiography, were also excluded. All patients provided written informed consent.

### Diabetes mellitus definition

Patients with DM were defined by one of the following criteria: (a) administration of any anti-diabetic medication; (b) diagnostic code of DM (International Classification of Diseases-11) in at least one hospital admission or previous physician-assigned DM diagnosis; (c) HbA1c value on admission measured above 6.5% (48 mmol/mol) [25].

### Measurement of biomarkers and metabolomic analyses

Venous blood sample was collected from all patients prior to their coronary angiography, following at least 8 h of fasting. The quantification of 2 ceramide species (24:0, 24:1) with their respective ratio and of short, medium- and long chain acylcarnitines in patients’ serum samples was achieved through targeted analytical methods already described by our group (Reversed Phase Liquid Chromatography tandem Mass Spectrometry, RPLC-MS/MS and  Hydrophilic Interaction Liquid Chromatography tandem  Mass Spectrometry, HILIC-MS/MS methods, respectively) [26, 27]. Finally, protein markers associated with CAD, namely apolipoproteins (apo) B and A1 were measured by quantitative protein nephelometry while adiponectin levels were quantified through enzyme-linked immunosorbent assay (ELISA).

### Study outcomes definition and follow-up

The primary outcome of this study was the composite of MACCE (cardiovascular death, stroke, myocardial infarction, major bleeding), repeat unplanned revascularization and cardiac-disease related hospitalizations. The vital status of patients was verified by telephonic follow-up or by accessing the Greek Civil Registration System. Furthermore, possible associations between metabolomic biomarkers and prediction of CAD severity were evaluated for the development of metabolomics-based prediction models and algorithms.

### Coronary artery disease complexity

Coronary artery disease complexity was assessed via the SYNTAX score, a precise angiographic tool for assessing the complexity of CAD [26]. Coronary angiography images of every patient were independently evaluated by 2 experienced interventional cardiologists, blinded to patient clinical characteristics and outcomes, and the SYNTAX score was calculated. Based on the SYNTAX score, patients were categorized into 3 subgroups, with: (i) low SYNTAX score (0–22), (ii) intermediate SYNTAX score (23–32) and (iii) high SYNTAX score (> 32) [28].

### Statistical analysis

Continuous variables are presented as means with standard deviations (SD) and compared using the Mann Whitney U test. Categorical variables are presented as frequencies with percentages and compared via the Pearson chi-square test. Univariable Cox regression analyses were performed to identify significant predictors of the study outcomes among the clinically relevant parameters (age, presence of acute coronary syndrome, smoking, estimated glomerular filtration rate (eGFR), history of heart failure (HF) and history of peripheral artery disease) and measured metabolomics data. These included glycated hemoglobin (HbA1C), apoA1, apoB, apoB/apoA1 ratio, adiponectin, peak high-sensitive Troponin T (hsTnT) and different acylcarnitine and ceramide species as well as their ratios. The rationale behind the measurement of specific acylcarnitine ratios was that they have been associated with increased atherosclerotic burden in a previous post-hoc analysis of the CorLipid trial [24]. Cox regression hazard models were then developed by forcing into the multivariable analysis clinically relevant baseline covariates and metabolomics biomarkers that were univariably significantly associated with the primary composite outcome. A p-value of less than 0.1 was used for assessing univariable significance.

Concerning CAD severity prediction, due to the non-parametric nature of the data, a bootstrapped multivariable linear regression model was similarly built to reveal independent predictors of higher SYNTAX score (dependent variable) after being adjusted for the aforementioned clinically relevant variables. Sub-analyses performed for the primary outcome and CAD complexity prediction to assess the predictive value of novel biomarkers only in patients with acute or chronic coronary syndrome. The generated adjusted hazard ratios (aHR) and beta coefficients are presented with their respective 95% confidence intervals (CI). Additionally, we performed receiver operating characteristic (ROC) analysis to investigate the sensitivity and specificity of these models as predictors of the primary outcome and CAD severity. A two-sided p-value of less than 0.05 was considered statistically significant. All analyses were conducted through the SPSS Statistics for Windows, Version 25.0 (Armonk, NY: IBM Corp) and Stata statistical software, release 13 (StataCorp).

## Results

### Baseline characteristics

Out of 1,024 patients enrolled in the CorLipid trial, 707 (69.8%) were excluded, and the current analysis involved 316 (30.2%) patients with comorbid DM. Of included patients, 176 (55.7%) underwent coronary angiography due to ACS [62 (19.6%) patients due to ST-Elevation Myocardial Infarction (STEMI), 59 (18.7%) patients due to Non-ST-Elevation Myocardial Infarction (NSTEMI), and 55 (17.4%) patients due to unstable angina], and 140 due to CCS (44.3%) [24]. Baseline demographical, clinical and laboratory characteristics of patients included in the present analysis are shown by the presence of acute or chronic coronary syndrome in Table 1. Comparison of all measured novel biomarkers between patients with ACS and CCS is summarized in Table 2.

In general, the baseline characteristics between the two groups were well-matched. The mean age of patients was 67 ± 11 years and the majority of patients were males (70.3%). Patients with ACS were more likely to smoke, have a history of chronic kidney disease and have a lower mean left-ventricular ejection fraction. Peak values of HsTnT were also much higher in the ACS group, while mean high-density lipoprotein values were significantly lower. Regarding clinically novel biomarkers, apoA1 was significantly lower in patients with ACS, and these patients also had a higher apoB/apoA1 ratio compared to patients with CCS.

**Table 1:** Baseline clinical and demographic characteristics and comorbidities in the CorLipid population by the presence of coronary syndrome

	All diabetic patients (n = 316)	Diabetic patients with ACS (N = 176)	Diabetic patients with CCS (N = 140)	P-value
Clinical characteristics, No. (%)				
Male gender	222 (70.3)	129 (73.3)	93(66.4)	0.18
STEMI	62 (19.6)	62 (35.2)	0 (0)	–
NSTEMI	59 (18.7)	59 (33.5)	0 (0)	–
Unstable angina	55 (17.4)	55 (31.3)	0 (0)	–
Smoking	129 (40.8)	87 (49.4)	42 (30)	**< 0.01**
Clinical parameters [mean (± SD) / Median (IQR = Q3-Q1)*]				
Age (years)	67 (11)	66 (13)	67 (10)	0.60
Body mass index (kg/m^2^)	29.4 (5.1)	28.8(4.9)	29.3 (5)	0.40
Estimated glomerular filtration rate by CKD-EPI (mL/min/1.73m^2^)	90 (39.3)	89 (40.7)	92.3 (36.9)	0.46
Left ventricular ejection fraction (%)	50 (10)	50 (11)	55 (9)	**< 0.01**
Total cholesterol (mg/dL)	155 (44)	156 (45)	155 (45)	0.86
Triglycerides (mg/dL)*	130 (1656)	130 (1656)	128 (1563)	0.90
High density lipoprotein (mg/dL)	41 (12)	40 (12)	44 (14)	**0.02**
Low density lipoprotein (mg/dL)	82 (36)	85 (38)	82 (35)	0.43
Fasting glucose (mg/dL)*	127 (416)	128 (399)	126 (380)	0.62
Glycated hemoglobin A1c (%)	7.3 (1.6)	7.1 (1)	7.2 (1.4)	0.34
Peak high-sensitivity cardiac troponin (ng/L)*	35(9996)	207(9996)	15(1582)	**< 0.01**
Medical histories, No. (%)				
Hypertension	237 (75)	127 (72.2)	110 (78.6)	0.19
Dyslipidemia	155 (49.1)	80(45.5)	75 (53.6)	0.15
Heart failure	9 (2.8)	7 (11.4)	2 (4.3)	0.17
Chronic kidney disease	26 (8.2)	20 (4.6)	6 (2.9)	**0.02**
Peripheral vascular disease	22 (7)	12 (6.8)	10 (7.1)	0.91
Atrial fibrillation	32 (10.1)	18 (10.2)	14 (10)	0.94
Prior stroke	12 (3.8)	5 (2.8)	7 (5)	0.31
Positive family history of CAD	47 (14.9)	29 (16.6)	18 (12.9)	0.35
Statin medication	185 (58.5)	97 (55.1)	88 (63.8)	0.12
Oral anticoagulant medication	45 (14.2)	31 (17.6)	14 (10.1)	0.061

Variables with p value < 0.05 were identified as significant variables and they are in bold

*Mean values and standard deviations are provided for variables with normal distribution, while median values and interquartile range (IQR) are provided for variables with non-normal distribution

**Table 2:** Baseline comparison of measured novel biomarkers in the CorLipid population by the presence of coronary syndrome

	All diabetic patients (n = 316)	Diabetic patients with ACS (N = 176)	Diabetic patients with CCS (N = 140)	P-value
Clinical parameters, Median (IQR = Q3-Q1)]*				
Acylcarnitine C4	41 (736)	40.6 (735)	42.5 (423)	0.87
Acylcarnitine C18:2	58 (264)	54.9 (263)	61.3 (151)	0.20
Acylcarnitine ratio C4/C18:2	0.72 (12.5)	0.76 (12.5)	0.69 (3.4)	0.41
ApoB	84.5 (412)	87.2 (412)	81.1 (152)	0.53
ApoA1	97.9 (193)	89.2 (185)	113 (168)	**< 0.01**
ΑpoB/ΑpoA1 ratio	0.79 (6.9)	0.84 (6.9)	0.70 (3.2)	**0.02**
Ceramide C24:0	7.5 (32.7)	7.4 (28.7)	7.7 (32.7)	0.80
Ceramide C24:1	3.2 (12.5)	3.4 (6.7)	3.1 (12.5)	0.08
Ceramide ratio C24:1/C24:0	0.45 (1.76)	0.45 (1.76)	0.42 (1.72)	0.18
Adiponectin	162 (215)	162 (207)	160 (214)	0.81

Variables with p value < 0.05 were identified as significant variables and they are in bold

*Median values and interquartile range (IQR = Q3–1) are provided for all those variables since they have non-normal distribution

### Clinical outcomes

After a median 2-year follow up period (IQR = 0.7 years), 36 of 316 (11.4%) study participants died. Of deaths, 30 (86.7%) were attributed to CV causes. The primary composite outcome occurred in 69 (21.8%) patients. This included 7 major bleeding events, 3 myocardial infarction, 4 strokes, 30 CV-related hospitalizations and 11 repeat unplanned coronary revascularization procedures. Univariable analyses yielded that acylcarnitine ratio C4/C18:2, apoB, HsTnT and HbA1c were associated with higher risk of the composite primary outcome in patients with CAD.

**Fig. 1:**
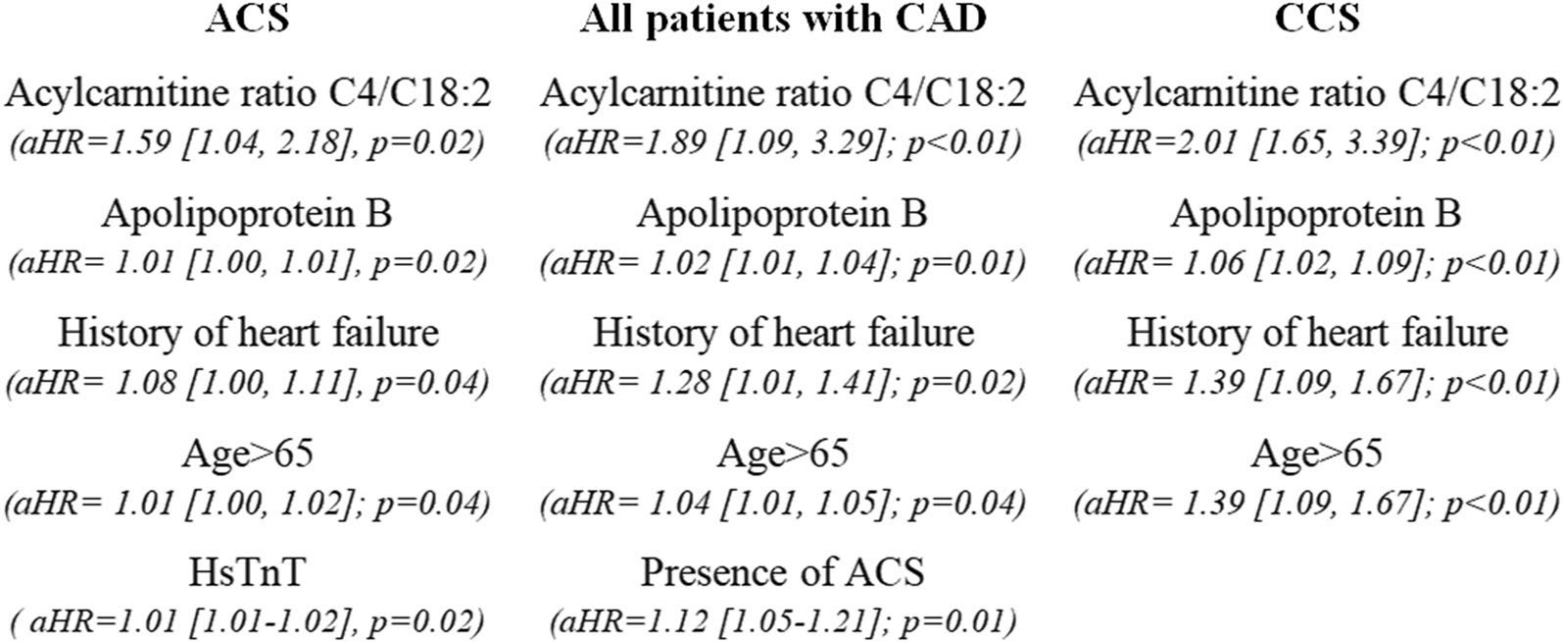
Significant parameters derived from the multivariable Cox regression models set for the prediction of the primary outcome. *ACS, acute coronary syndrome; CCS, chronic coronary syndrome; aHR, adjusted hazard ratio; CAD, coronary artery disease

**Fig. 2:**
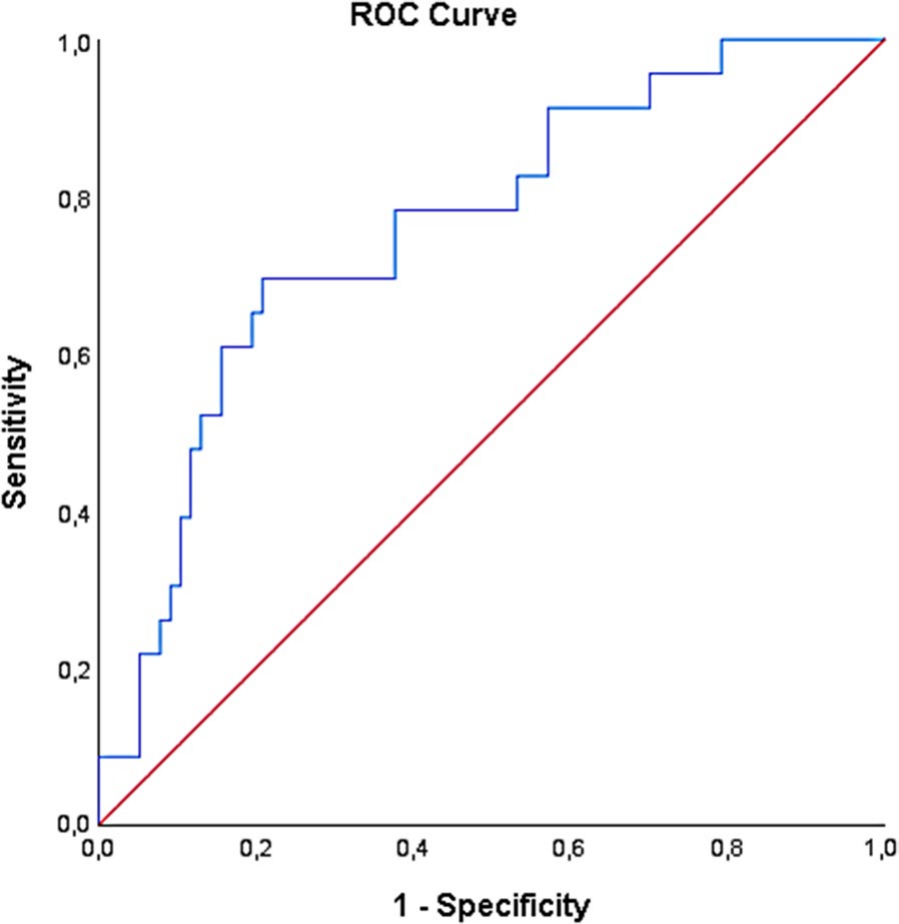
Receiver operating characteristic (ROC) analysis on the predictive capacity of the created multivariable Cox regression model for the occurrence of adverse clinical outcomes in patients with CAD and DM. ROC analysis on the predictive capacity of the created multivariable Cox regression model for the identification of the hazard for the primary composite outcome of major adverse cardiovascular or cerebrovascular events (MACCE; cardiovascular death, stroke, myocardial infarction, major bleeding), repeat unplanned revascularization and cardiovascular hospitalizations (area under the curve [AUC]: 0.76, 95% CI 0.60–0.87; Chi-square = 23.979, P = 0.017)

These biomarkers were then forced into the multivariable Cox regression model along with clinically relevant baseline covariates which were invariably significant (presence of ACS, age > 65, history of HF, history of peripheral artery disease). According to this multivariable analysis, higher levels of acylcarnitine ratio C4/C18:2 (aHR = 1.89 [1.09, 3.29]; p < 0.01), and apoB (aHR = 1.02 [1.01, 1.04]; p = 0.01), as well as history of HF (aHR = 1.28 [1.01, 1.41]; p = 0.02), age > 65 (aHR = 1.04 [1.01, 1.05]; p = 0.04) and presence of ACS (aHR = 1.12 [1.05–1.21]; p = 0.01) were independent predictors of more frequent MACCE/repeat revascularization/cardiovascular hospitalization occurrence (Fig. 1). The ROC analysis for the prediction of the primary outcome in patients with CAD according to the developed model is depicted in Fig. 2 (area under the curve [AUC]: 0.76, 95% CI 0.60–0.87).

Sub-analysis in patients with ACS showed that history of HF, age > 65, higher levels of acylcarnitine ratio C4/C18:2 and apoB as well as higher levels of peak HsTnT were independently associated with the primary outcome (Fig. 1). In patients with CCS, acylcarnitine ratio C4/C18:2, apoB, history of HF, and age > 65 predicted independently the occurrence of the primary composite outcome (Fig. 1).

**Fig. 3:**
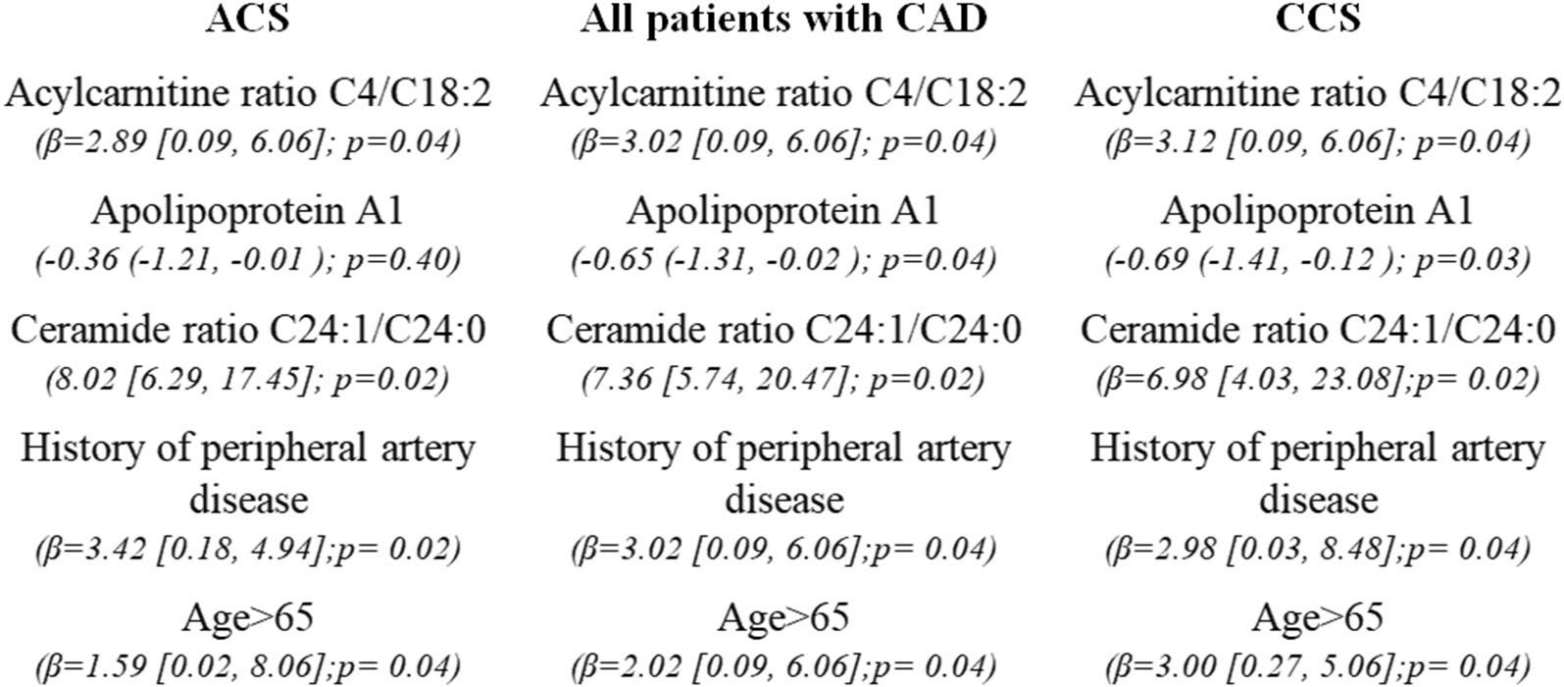
Significant parameters derived from the multivariable linear regression models set for the prediction of coronary artery disease complexity. *ACS, acute coronary syndrome; CCS, chronic coronary syndrome; β; adjusted beta coefficient; CAD, coronary artery disease

**Fig. 4:**
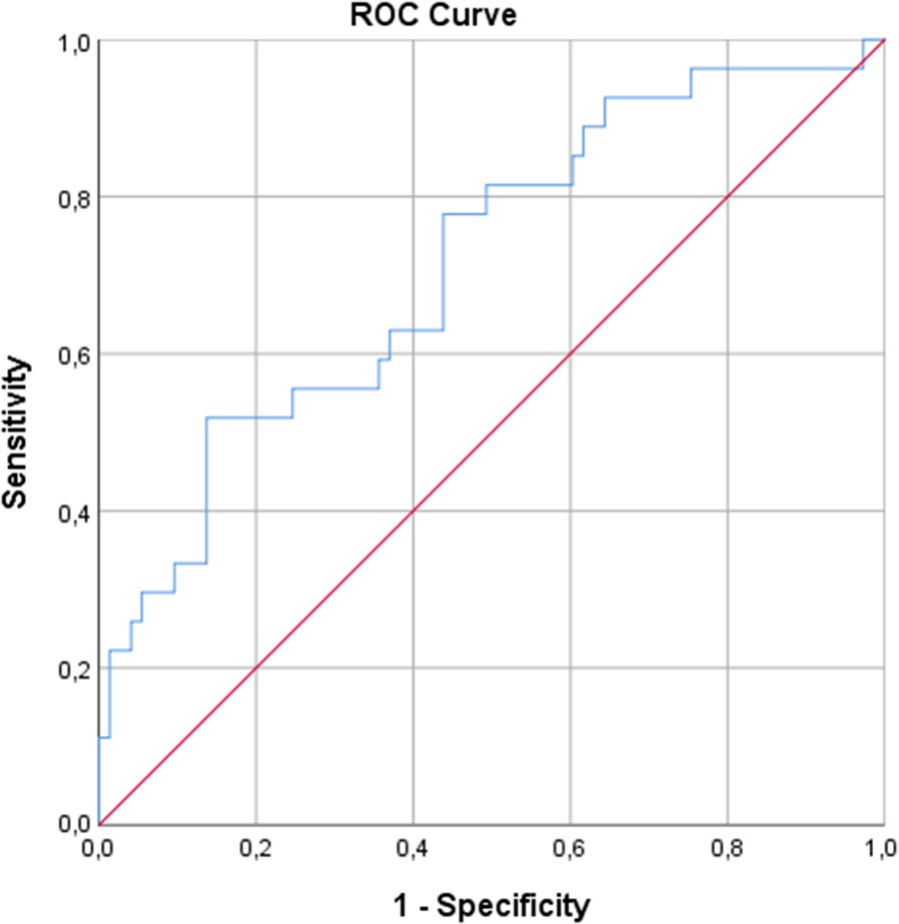
Receiver operating characteristic (ROC) analysis on the predictive capacity of the created multivariable linear regression model for the prediction of coronary artery disease complexity in patients with CAD and DM. ROC analysis on the predictive capacity of the developed bootstrapped multivariable linear regression model for the prediction of coronary artery disease complexity (AUC: 0.71, 95% CI 0.60–0.83; R-square = 0.220, Durbin-Watson = 2.131, P = 0.002)

As far as CAD complexity is concerned, univariable linear regression analyses showed that higher levels of acylcarnitine C4, acylcarnitine ratio C4/C18:2 and ceramide ratio C24:1/C24:0, age > 65, and history of HF were significantly associated with higher SYNTAX score, whereas higher ApoA1 levels were associated with decreased SYNTAX score. The developed multivariable regression model, detailed in Fig. 3, demonstrated that ceramide ratio C24:1/C24:0 (adjusted β = 7.36 [5.74, 20.47]; p = 0.02), acylcarnitine ratio C4/C18:2 (adjusted β = 3.02 [0.09 to 6.06]; p = 0.04), history of peripheral artery disease (β = 3.02 [0.09, 6.06]; p = 0.04), and age > 65 (β = 2.02 [0.09, 6.06]; p = 0.04) were independently and positively associated with the SYNTAX score, while ApoA1 (adjusted β= − 0.65 [− 1.31, − 0.02]; p = 0.04) independently predicted decreased SYNTAX score. The ROC analysis performed for the model set to predict CAD complexity is shown in Fig. 4 (AUC: 0.71; 95% CI 0.60–0.83).

Sub-analyses in ACS group showed that acylcarnitine ratio C4/C18:2 (β = 2.89 [0.09, 6.06]; p = 0.04), ceramide ratio C24:1/C24:0 (8.02 [6.29, 17.45]; p = 0.02), history of peripheral artery disease (β = 3.42 [0.18, 4.94]; p = 0.02), and age > 65 (β = 1.59 [0.02, 8.06]; p = 0.04) still predicted independently the SYNTAX score, whereas ApoΑ1 (− 0.36 (− 1.21, − 0.01); p = 0.40) was an independent predictor of decreased SYNTAX score. Acylcarnitine ratio C4/C18:2 (β = 3.12 [0.09, 6.06], p = 0.04), ceramide ratio C24:1/C24:0 (β = 6.98 [4.03, 23.08], p = 0.02), history of peripheral artery disease (β = 2.98 [0.03, 8.48], p = 0.04), age (β = 3.00 [0.27, 5.06], p = 0.04), and ApoA1 (β= − 0.69 [− 1.41, − 0.12]; p = 0.03) were also independent predictors of CAD complexity in patients with CCS.

## Discussion

This post hoc analysis of the CorLipid clinical trial based on a well-selected population of hospitalized patients with DM and suspected or known history of comorbid CAD who underwent coronary angiography, showed that higher levels of apoB, acylcarnitine ratio C4/C18:2, and HsTnT were associated with higher risk for the primary composite outcome of MACCE, repeat unplanned revascularization and cardiac-disease related hospitalizations. Patients with higher values of ceramide ratio C24:1/C24:0 and acylcarnitine ratio C4/C18:2 had increased CAD complexity by invasive angiography, whereas patients with higher apoA1 levels had lower CAD complexity. To our knowledge, this is the first study attempting to assess such a large number of novel metabolomics biomarkers in patients with CAD and DM, thereby enabling both clinical outcome prediction of the comorbidity and also non-invasive identification of diabetics with high likelihood of severe CAD.

Concerning apolipoproteins, our findings concur with recent observational data suggesting that apoB lipid particles play a central role in atherogenic process and cardiovascular risk [29, 30]. This can be explained by laboratory data showing that apoB particles within endothelium constitute the key element of endothelium damage. Trapping of apoB deposits atherogenic cholesterol within the endothelium which causes injury to the arterial wall [31]. Furthermore, our results indicate that apoB could be considered as a useful predictor of cardiovascular mortality and morbidity in diabetic patients [32, 33]. This is consistent with the 2019 ESC Guidelines on Dyslipidaemias, which highlight that apoB provides an accurate estimate of the total concentration of atherogenic particles under all circumstances Of note, considering the potential inaccuracy of LDL-C in dyslipidaemia among patients with DM, guidelines highlight that apoB is superior to low density lipoprotein cholesterol (LDL-C) in estimating the atherosclerotic cardiovascular risk in this population and therefore should be the preferred measurement [34]. On the contrary, apoA1, a major protein component of high-density lipoprotein cholesterol (HDL-C) is strongly and inversely correlated to the risk of atherosclerosis and progression of vascular diseases [29]. Molecular research in diabetic mice, as well as clinical trials including diabetic individuals have proven that apoA1 promotes atherosclerosis regression, while decreased levels of this biomarker are blamed for increased incidence of atherosclerotic disease [30, 31]. The main pathophysiologic mechanism behind the cardioprotective effects of apoA1 is its role as the main initiator and driver of “the reverse cholesterol transport” [35]. Additionally, ApoA1 is capable of stimulating endothelial production of nitric oxide and release of prostacyclins, while it also manifests anti-oxidant and anti-inflammatory effects [36]. Another remarkable finding of our analysis respecting apolipoproteins is the comparison of their baseline values between patients with ACS and CCS. Patients with ACS seemed to have significantly lower baseline values of apoA1 and higher apoB/apoA1ratio. This implies that deregulation of apolipoproteins metabolism, except for predicting long-term cardiovascular risk, is associated with ongoing acute cardiovascular events in patients with DM [37]. With regard to HsTnT, previous studies have also highlighted it as a sensitive and easily quantifiable biomarker useful in determining the probability of MACCE in patients with acute coronary syndromes [38, 39]. A recent, large observational study showed that adding hs-cTnI to the Framingham Risk Score provided incremental prognostic benefit [40].

Our results regarding acylcarnitines indicate that high serum levels of these intermediate products of metabolism are associated with increased risk of cardiovascular events. Being lipidemic biomarkers involved in fatty acid metabolism, acylcarnitines are crucial for normal cell function as they regulate mitochondrial β-oxidation and maintain balance between free and esterified coenzyme A [41]. Thus, high acylcarnitine levels indicate disturbed fatty acid metabolism which is a metabolic substrate for atherosclerosis development, plaques’ vulnerability, and subsequent adverse events [42]. Our findings are similar to other studies showing increased cardiovascular risk in patients with elevated acylcarnitine levels [41, 43–45]. However, there is scarcity of data focusing only on patients with DM and associating specific acylcarnitine species with cardiovascular outcomes this population [24, 41]. Besides, this study adds to the existing literature regarding the relation of acylcarnitines with CAD complexity, and highlights the potential dynamic relationship between acylcarnitines and the severity of CAD. These along with recent research of our team based on observational data also derived from the CorLipid trial [24], are among the first to suggest that acylcarnitine levels reflect CAD severity in diabetic patients and might play a role in future patients’ stratification strategies. On the other hand, ceramides -members of the sphingolipid family- are well-established metabolomics biomarkers and have been already presented as potently robust predictors of cardiovascular disease and adverse outcomes [45, 46]. On a pathophysiologic basis, these bioactive lipid mediators, have signalling roles in apoptosis, cellular stress and inflammation and are also implicated in endothelial angiogenesis and vasoconstriction [47]. Higher serum concentrations of particular species, such as ceramides C16:0, C18:0, 24:0 and 24:1, have been linked with large thrombus burden and advanced CAD complexity in patients with acute coronary syndrome [48]. Elevated concentrations of circulating ceramides have been also associated with atherosclerotic plaque formation, ischemic cardiomyopathy, acute coronary events, insulin resistance and type 2 DM [49]. Current research supports the clinical utility of serum ceramides as efficient risk stratifiers for atherosclerotic severity [50].

Our findings could have an impact on featuring metabolomic profiling as a useful tool in routine clinical practice in patients at high risk of CAD. Validation of novel metabolomics biomarkers, offering additional prognostic value to traditional clinical and laboratory parameters, could aid clinicians in further risk-stratification of patients with DM. This could facilitate the creation of personalized predictive algorithms, thereby expanding the concept of cardiovascular precision medicine in every-day practice [51].

Our study should be interpreted in the context of its limitations. First, this is an observational analysis, prone to unrecognized biases inherent to such studies, despite multivariable adjustment. Secondly, in this post-hoc study of diabetic patients we included only a part of the patients enrolled in the CorLipid trial, which might indicate selection bias. Third, the data used for the current analysis derive from a relatively limited sample of Greek patients with suspected or known CAD and DM and hence the conclusions drawn warrant validation in larger clinical trials, including other European ethnicities and races to account for inherent variability of different patient populations. Fourth, we were not able to adjust for dietary and lifestyle parameters, which may impact our metabolomics results. Lastly, we could not account for specific anti-diabetic medication prescribed in our participants which might influence patients’ metabolic profile, since there was no such available data.

## Conclusions

In patients with CAD and comorbid DM who underwent emergency or elective coronary angiography, increased levels of apoB and acylcarnitine ratio C4/C18:2 were independent predictors of more frequent adverse clinical outcomes. Additionally, acylcarnitine ratio C4/C18:2, along with ceramide ratio C24:1/C24:0 were associated with high-complexity CAD, whereas higher levels of apoA1 were linked with lower SYNTAX score. Thus, metabolomic phenotyping and novel metabolomics-based biomarkers could be recruited to predict clinical outcomes and assess the complexity of CAD, thereby facilitating personalized risk-stratification and clinical decision making in diabetic patients. Larger trials are needed to confirm these results and validate them in different diabetic populations.

## Data Availability

Data are available from Georgios Sianos (e-mail: gsianos@auth.gr) upon reasonable request and with permission of AHEPA University Hospital.

## Abbreviations

(a)HR: (adjusted) hazard ratio
Apo A1: Apolipoprotein A1
Apo B: Apolipoprotein B
CAD: Coronary artery disease
CI: Confidence interval
DM: Diabetes mellitus
Hs-cTnT: High-sensitivity cardiac troponin test
MACCE: Major adverse cardiovascular or cerebrovascular events
